# Editorial: Autoimmune diseases and cardiovascular health

**DOI:** 10.3389/fcvm.2026.1993489

**Published:** 2026-09-24

**Authors:** Nicolas Vuilleumier, Guoping Li, Sabrina Pagano

**Affiliations:** 1 Division of Laboratory Medicine, Diagnostic Department, Geneva University Hospitals, Geneva, Switzerland; 2 Department of Medicine, Medical Faculty, Geneva University, Geneva, Switzerland; 3 Department of Anesthesiology, Mass General Brigham and Harvard Medical School, Boston, MA, United States

**Keywords:** autoantibodies, autoimmune diseases, cardiovascular risk, chronic inflammation, subclinical atherosclerosis

Over the past decades, the relationship between the immune system and cardiovascular (CV) disease has evolved from a peripheral hypothesis to a central theme in modern medicine. While traditional CV risk factors remain foundational, they do not fully account for the cardiovascular burden observed in many patient populations, particularly in patients with autoimmune disease (AID), where the increased CV risk is independent of classical CV determinants and where conventional risk scores perform sub-optimally in risk stratification (1).

This observation has prompted a shift in perspective. Rather than viewing cardiovascular complications merely as secondary consequences of systemic disease, there is increasing recognition that immune-mediated mechanisms directly contribute to cardiovascular pathologies. Chronic inflammation, immune dysregulation, and autoantibody production have all been shown to play active roles in shaping CV disease and its associated risks, paving the way for innovative therapeutic intervention (2–5).

In this context, the present Special Issue on *Autoimmune Diseases and Cardiovascular Health* aimed to explore the intersection between autoimmunity and cardiovascular disease from multiple complementary perspectives. The six contributions included in this issue—ranging from prospective multicenter investigations to meta-analyses and a case report—reflect both the diversity of approaches and the rapid evolution of the field. Collectively, they offer a coherent, albeit still incomplete, picture of how autoimmunity intersects with CV pathology and risk stratification.

One of the recurring themes is the presence of subclinical atherosclerosis and cardiac involvement in AID, particularly rheumatoid arthritis (RA). Advanced imaging techniques, especially cardiac magnetic resonance imaging, one of the studies included in this issue shows that in RA asymptomatic patients, the levels of myocardial fibrosis and ventricular dysfunction correlate with disease severity, suggesting that cardiac involvement mirrors systemic inflammatory activity.

This raises practical questions: how often do we underestimate cardiovascular involvement simply because it remains clinically silent and how could it be better captured? The meta-analyses included in this issue point toward a possible autoantibodies-based approach, or in another words, on a biomarker-based approach. This work extends previous findings in AID to the specific context patient suffering from asymptomatic AID where various biological autoimmune signatures appear to be associated with an increased risk of future CV events. This reinforces the idea that immune dysregulation is not merely a bystander but may precede—and possibly drive—cardiovascular disease. From a clinical perspective, this opens the door to earlier and potentially more refined risk stratification.

The role of autoantibodies emerges as a particularly compelling thread across several contributions. Traditionally viewed as hallmarks of autoimmune diseases, autoantibodies are increasingly being recognized for their potential pathogenic and prognostic roles in CV conditions and could therefore represent valuable biomarker candidates. In this context, the association between autoantibodies targeting G protein–coupled receptors—such as endothelin receptor type A and endothelin-1 receptor—and myocardial infarction in individuals recruited during any acute myocardial infarction without known AID is especially striking. It shows that features of biological AID are detectable in patients without AID and predict future CV events, transposing the conventional boundaries of autoimmunity to broader patient populations.

The heterogeneity of immune-mediated vascular involvement is further illustrated by the case report describing antineutrophil cytoplasmic antibody–associated disease affecting large vessels. While such presentations remain relatively uncommon, they serve as a reminder that autoimmune processes can involve the vascular system at multiple levels, from the microcirculation to major arteries. Recognizing these patterns is essential, particularly when clinical presentations fall outside the expected spectrum.

Two contributions in this Special Issue also draw attention to emerging autoantibodies with potential clinical relevance, notably anti-apolipoprotein A-I antibodies. These antibodies are shown to independently predict cardiovascular mortality in patients with rheumatoid arthritis, beyond established risk factors. Their significance may extend beyond classical autoimmune diseases. In conditions characterized by chronic immune activation—such as human immunodeficiency virus infection- their presence could reflect a broader state of immune imbalance with cardiovascular consequences. Such observations further blur the distinction between autoimmune and non-autoimmune contexts.

Taken together, the studies presented here converge toward a few key messages.

First, autoimmune diseases should be considered as high-risk CV conditions *per se*. Second, advanced technological modalities (including radiology and blood autoimmune biomarkers) provide valuable tools to detect early and otherwise unrecognized cardiac involvement. Third, immune-mediated cardiovascular risk is not confined to classical autoimmune diseases but extend to a wider spectrum of conditions marked by immune dysregulation.

At the same time, many questions remain. The precise mechanisms linking autoantibodies to CV injuries are still incompletely understood. Whether modulating immune pathways can translate into meaningful cardiovascular risk reduction is another critical issue. More broadly, if autoantibodies qualify as valuable biomarkers candidates for risk stratifications purposes by (i) preceding the development of clinically overt diseases and/or complications by many years (6), and (ii) fulfilling the usual analytical requirements (i.e., adequate analytical sensitivity and specificity, availability, low cost, minimally invasive and easily up scalable), incorporating them into routine clinical practice for CV assessment will require careful consideration of the population, targeted, feasibility, standardization, and added values compared to state-of-the-art and actionable biomarkers in the field (ie. high sensitive troponins, brain natriuretic peptides).

In many ways, the field is at a turning point. The traditional separation between cardiology and immunology is becoming increasingly difficult to maintain, as evidence accumulates that these systems are deeply interconnected. The studies gathered in this Special Issue reflect this shift and highlight the need for closer integration across disciplines.

Ultimately, a more nuanced understanding of immune-mediated cardiovascular risk may lead to more personalized approaches to prevention and treatment. Moving in that direction will require not only further research but also a willingness to rethink established frameworks. The contributions presented in this Special Issue represent an important step in that direction.
